# Phages limit the evolution of bacterial antibiotic resistance in experimental microcosms

**DOI:** 10.1111/j.1752-4571.2011.00236.x

**Published:** 2012-01-13

**Authors:** Quan-Guo Zhang, Angus Buckling

**Affiliations:** 1 State Key Laboratory of Earth Surface Processes and Resource Ecology and MOE Key Laboratory for Biodiversity Science and Ecological Engineering, Beijing Normal UniversityBeijing, China; 2 Biosciences, University of ExeterCornwall Campus, Penryn, UK

**Keywords:** coevolution, fitness cost, immigration, mutator bacteria, phage therapy

## Abstract

The evolution of multi-antibiotic resistance in bacterial pathogens, often resulting from *de novo* mutations, is creating a public health crisis. Phages show promise for combating antibiotic-resistant bacteria, the efficacy of which, however, may also be limited by resistance evolution. Here, we suggest that phages may be used as supplements to antibiotics in treating initially sensitive bacteria to prevent resistance evolution, as phages are unaffected by most antibiotics and there should be little cross-resistance to antibiotics and phages. *In vitro* experiments using the bacterium *Pseudomonas fluorescens*, a lytic phage, and the antibiotic kanamycin supported this prediction: an antibiotic–phage combination dramatically decreased the chance of bacterial population survival that indicates resistance evolution, compared with antibiotic treatment alone, whereas the phage alone did not affect bacterial survival. This effect of the combined treatment in preventing resistance evolution was robust to immigration of bacteria from an untreated environment, but not to immigration from environment where the bacteria had coevolved with the phage. By contrast, an isogenic hypermutable strain constructed from the wild-type *P. fluorescens* evolved resistance to all treatments regardless of immigration, but typically suffered very large fitness costs. These results suggest that an antibiotic–phage combination may show promise as an antimicrobial strategy.

## Introduction

The rapid evolution of antibiotic resistance in bacterial pathogens (Cohen 1992; Levy and Marshall 2004; Martinez et al. 2007) and a continued decline in the supply of new antibiotics (Donadio et al. 2010) have created an urgent need for novel antimicrobial approaches. Combined use of multiple antibiotics is increasingly employed to clear infections, which can also limit resistance evolution to a greater extent than single antibiotic therapy (Bonhoeffer et al. 1997; Lipsitch and Levin 1997; Chow and Yu 1999; Yeh et al. 2009; zur Wiesch et al. 2011). However, multi-antibiotic resistance in bacterial pathogens, resulting from either single mutations (or horizontally transferred genes) conferring cross-resistance to several antibiotics or accumulation of multiple mutations conferring resistance to single antibiotics, has frequently been observed (McCormick et al. 2003; Wright et al. 2006).

Recent years have seen a resurgent interest in the therapeutic use of lytic bacteriophages (phages) to treat pathogenic bacterial infections (Levin and Bull 1996; Barrow and Soothill 1997; Alisky et al. 1998; Chanishvili et al. 2001; Summers 2001; Biswas et al. 2002; Bull et al. 2002, 2010; Kutateladze and Adamia 2010; Monk et al. 2010; Pirnay et al. 2011). However, the dilemma for antibiotic use is also faced by phage therapy: bacteria may readily evolve resistance to phages that attack them (Luria and Delbruck 1943; Smith et al. 1987; Alisky et al. 1998; Chanishvili et al. 2001; Summers 2001). While phages may also evolve rapidly and adapt to infect resistant bacteria (Alisky et al. 1998; Thiel 2004; Brockhurst et al. 2007), subsequently resulting in coevolutionary arms races between bacteria and phages, the therapeutic significance of this is currently unclear.

Simultaneous use of phages and antibiotics may help to minimize the chance of resistance evolution for the same reasons as treatment with multiple antibiotics (Bonhoeffer et al. 1997; Lipsitch and Levin 1997; Chow and Yu 1999; Yeh et al. 2009; zur Wiesch et al. 2011). First, the combined treatment is likely to reduce bacterial population size, and hence mutation supply rate, relative to single treatments. Consistent with this view, combined treatments of antibiotics and phages have been shown to be better or equally good at decreasing bacterial abundance compared with antibiotics or phages alone (Alisky et al. 1998; Chanishvili et al. 2001; Comeau et al. 2007). Second, there is typically little cross-resistance to antibiotics and phages (Chanishvili et al. 2001; Kutateladze and Adamia 2010); hence, simultaneous multiple mutations are required for bacterial resistance evolution.

Here, we experimentally address the effect of combined antibiotic–phage treatment on bacterial resistance evolution using laboratory populations of a bacterium *Pseudomonas fluorescens* SBW25. By making use of a convenient *in vitro* experimental system, we were able to carry out a relatively extensive study, and the central results should apply to a broad range of systems. We treated bacterial cultures with an antibiotic (kanamycin) and a lytic phage (SBW25Φ2), separately or simultaneously, and then examined the probability of bacterial population survival (which indicates resistance evolution) and the fitness of bacterial populations that survived the treatments. We also examined whether the antimicrobial treatments were robust to bacterial immigration which has previously been suggested to foster adaptive evolution in stressful (such as under antibiotic treatment) environments (Holt and Gaines 1992; Perron et al. 2007). Furthermore, the experiment was replicated using both the wild-type *P. fluorescens* SBW25 and an isogenic mutator SBW25*mutS* so that we were able to address the consequences of bacterial hypermutability for resistance evolution.

## Materials and methods

### Strains and culture conditions

We used two bacterial strains, *P. fluorescens* SBW25 (Rainey and Bailey 1996) and an isogenic *mutS* knockout (mutator) of the wild-type SBW25 which has a approximately 100-fold higher mutation rate (Pal et al. 2007) and a lytic bacteriophage SBW25Φ2 (Buckling and Rainey 2002). Bacteria and phages were grown at 28 (±0.2)°C in microcosms of 200 μL of 0.1 KB medium (M9 salt solution supplemented with 1 g L^−1^ glycerol and 2 g L^−1^ proteose peptone no. 3) in 96-well microplates. A bactericide kanamycin (8.0 mg L^−1^) was used in this study as an antibiotic, which kills over 99% of the ancestral bacterial cells within 8 h under our culture conditions.

### Selection experiment

We first examined the effect of the antibiotic and the phage on bacterial population survival that indicates resistance evolution (as populations that did not acquire resistance would have gone extinct because of serial dilutions). Either the wild-type or the mutator bacteria were grown under four treatments: (*i*) control: antibiotic-free and phage-free, (*ii*) antibiotic (alone), (*iii*) phage (alone), and (*iv*) combined antibiotic–phage treatment.

We then investigated whether immigration of bacteria from habitable environments (source environments; 0.1 KB medium) can increase the probability of bacterial resistance evolution. For bacteria under the antibiotic treatment, we considered the effect of immigration of bacteria from 0.1 KB environment. For populations under the combined antibiotic–phage treatment, we considered two types of source environments: 0.1 KB and the phage-treatment environment. Therefore, three more treatments were set up: (*v*) antibiotic treatment with immigration of bacteria (from 0.1 KB environment), (*vi*) antibiotic–phage treatment with immigration of bacteria (from 0.1 KB environment), and (*vii*) antibiotic–phage treatment with immigration of bacteria/phage (from phage-treatment environment). We also set up three types of source populations to ensure that every sink microcosm under treatment *v*, *vi*, or *vii* had an independent source microcosm.

Twenty-four replicate microcosms were grown under each treatment, yielding a total of 240 microcosms (24 replicates × 10 types of populations, including the source populations for treatment *v*, *vi,* and *vii*). Each microcosm (200 μL of 0.1 KB medium) was initially inoculated with approximately 10^6^ stationary-phase bacterial cells (from a culture of the ancestral bacteria grown for 48 h), and, for microcosms with phage treatment, approximately 10^4^ ancestral phage particles. Cultures were propagated for 12 serial transfers, one transfer every 2 days. At every transfer, 2 μL (1%) of culture from each microcosm was transferred to 198 μL of fresh medium, and for each microcosm with phage treatment, approximately 10^2^ ancestral phage particles were introduced to ensure the persistence of the phage; immigration was then carried out immediately by transferring 1% of each source microcosm to the recipient microcosm. At each transfer, we used four microplates, and in each microplate, we grew 60 microcosms (with the 60 central wells): 10 types of populations × six replicates. Bacterial growth was measured as optical density (OD) at 600 nm before each transfer (there is a linear relationship between OD and bacterial density for OD values ranging from 0.1 to 0.5; bacterial density for 1 OD unit: ∼3 × 10^9^ cells mL^−1^). Based on measurements of bacterial colony counts of a subset of microcosms (Data S1), we considered populations with OD ≥ 0.05 (with bacterial density > ∼10^8^ cells mL^−1^) as surviving (with resistance acquired) and populations with an OD < 0.05 as nonviable (without resistance).

### Fitness assays

Fitness of populations surviving the different treatments was measured by competition assays. At the end of the experiment (transfer 12), up to six bacterial populations with OD ≥ 0.05 were randomly chosen for each treatment (for any treatment with <6 populations surviving, all populations with OD ≥ 0.05 were chosen), of which fitness relative to the ancestor in antibiotic-free and phage-free environment was measured. To remove phages from the cultures, we transferred 2 μL of culture from each microcosm to 198 μL of fresh medium with 0.15% Virkon (a commercially available disinfectant; Antec International, Sudbury, England) and left static for 24 h (this procedure left the bacteria viable and completely phage free), of which 2 μL was added to 198 fresh medium and grown for 24 h to give a phage-free and Virkon-free culture (Morgan et al. 2005; Lopez-Pascua et al. 2010), and the phage-free and Virkon-free cultures were used for competition assays. All the chosen populations (whether or not containing phages) and the ancestral bacteria were exposed to the Virkon-treatment procedure to minimize its influence on measurement of fitness of different populations. One microliter of each of these cultures (approximately 10^6^ cells) was inoculated into fresh medium, together with 1 μL of *P. fluorescens* SBW25EeZY6KX (Bailey et al. 1995) culture, and grown for 48 h, the duration of a transfer of the selection experiment. Initial and final densities of bacteria were measured by plating diluted cultures onto KB agar plates supplemented with X-gal (40 μg mL^−1^) on which SBW25 produces yellow colonies and SBW25EeZY6KX (with a *lacZY* insert) produces blue colonies. Fitness of SBW25 (*W*) relative to SBW25EeZY6KX was calculated from the ratio of the estimated Malthusian parameter, *m* = ln (*N*_f_/*N*_0_), with *N*_0_ and *N*_f_ the relevant initial and final densities (Lenski et al. 1991); thus *W* = *m*_SBW25_/*m*_SBW25EeZY6KX_. We then calculated fitness of the evolved SBW25 populations relative to the ancestor as an estimate of the selection coefficient (*S*) by subtracting *W* of the ancestral SBW25 from *W* of each of the evolved SBW25 population (i.e., *S* = *W*_evovled_−*W*_ancestor_; Lenski et al. 1991).

We also isolated several types of resistant mutants from cultures of the ancestral bacteria using selective agar plates (for methods to isolate resistant mutants, see Data S1, *Mutation rates*), referred to as ‘first-generation’ resistant mutants, and measured fitness of these mutants. For either the wild-type or the mutator bacteria, we obtained six antibiotic-resistant colonies, six phage-resistant colonies (resistant to the ancestral phage), and six double-resistance colonies (resistant to both the antibiotic and the ancestral phage) from cultures of the ancestral strains, of which fitness difference from the ancestor (selection coefficient) in antibiotic-free and phage-free environment was measured by competition experiments.

One-sample *t*-tests were used for the difference of selection coefficient (*S*) values of populations under each treatment from a null hypothesis value of 0. Two-sample *t*-tests were used for the difference in *S* values between populations under different treatments (note that we did not use anova for the effects of antibiotic and phage treatments because of the small sample size of certain treatment: only one wild-type bacterial population survived the combined antibiotic–phage treatment). We determined whether fitness costs of resistance decreased or increased during the selection experiment by one-sample *t*-tests on *S* values of the evolved populations, with the mean *S* value of the first-generation mutants of relevance (antibiotic-, phage-, or double-resistant mutants) as the expected value.

## Results

### The antibiotic and the phage synergistically prevented resistance evolution in the wild-type bacteria

**Figure 1:**
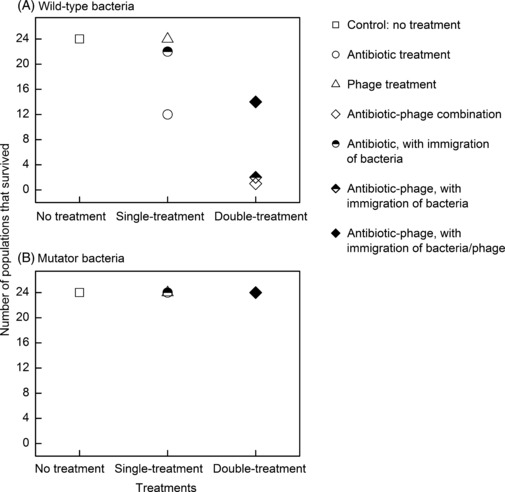
The number of bacterial populations that survived at the end of the experiment (of a total of 24 populations) under each antimicrobial treatment for the wild-type (A) or mutator (B) bacterial strain.

The bacterium *P. fluorescens* SBW25 can evolve resistance by *de novo* mutation to both the antibiotic kanamycin and the phage SBW25Φ2. The wild-type bacterial strain had a mutation rate of approximately 10^−7^ per cell per generation to either kanamycin resistance or phage resistance (resistance to the ancestral phage), and there was no evidence for cross-resistance against the antibiotic kanamycin and the phage (Data S1; Figure S1). Neither the phage nor the antibiotic alone was a very effective antimicrobial agent. The phage could not control bacterial population survival: all wild-type bacterial populations under the phage treatment survived (Fig. 1A), as was the case when populations were grown in the control (antibiotic free and phage free) environment. Treatment with the antibiotic alone significantly reduced the probability of bacterial survival, with 12 of 24 populations surviving (compared with the control, Fisher’s exact test, *P* < 0.001). Crucially, combining the antibiotic and the phage had a synergistic effect on bacterial survival, with only one of 24 populations surviving (lower than the control, phage, or antibiotic treatment, *P* < 0.001; Fig. 1A; see raw data in Figure S3).

### The effect of immigration on resistance evolution in the wild-type bacteria depended on the source population

In our experiment with the wild-type bacteria, the probability of resistance evolution in the antibiotic environment was dramatically increased by immigration of bacteria from the control (antibiotic free and phage free) environment, with 22, compared with 12, populations surviving (*P* = 0.003; Fig. 1A). However, immigration of bacteria from the control environment did not affect population survival under the combined antibiotic–phage treatment (*P* = 1.00; Fig. 1A).

We then explored the impact of migration on bacterial survival under the combined antibiotic–phage treatment, where the source populations were also exposed to phage treatment. We anticipated that bacterial growth in sink microcosms might be further reduced by the immigrant bacteria/phages through ‘apparent competition’ (Holt and Barfield 2009; Ricklefs 2010), particularly if the immigrant phages were more infective as a result of more extensive coevolution in the relatively benign source environment (Forde et al. 2004; Lopez-Pascua et al. 2010). However, we found that resident phages were as infective as immigrant phages (Data S1; Figure S2), and immigration in this context greatly increased bacterial survival (*P* < 0.001; Fig. 1A).

### The mutator bacteria survived all types of treatments

Compared with the wild-type bacteria, the mutator had a approximately 10-fold higher mutation rate to kanamycin resistance (approximately 10^−6^ per cell per generation) and a approximately 10^4^-fold higher mutation rate to phage resistance (approximately 10^−3^; Data S1 and Figure S1). All populations of mutator bacteria survived every treatment (Fig. 1B; see raw data in Figure S4), and thus, we observed no effect of immigration. Measurement of phage densities suggests that the phages, although able to attack the ancestral mutator bacteria, failed to grow within five transfers (Data S1). It is likely that the mutator bacteria evolved very quickly to evade phage infection and the phages were unable to persist.

### Fitness of surviving populations differed among antimicrobial treatments

Antibiotic-resistant bacteria often have a lower fitness than their sensitive counterparts when grown in antibiotic-free environment. Such fitness costs can result in selection against, and subsequent loss of, antibiotic resistance when antibiotic use is terminated (Bonhoeffer et al. 1997; Levin et al. 2000; Ward et al. 2009; Andersson and Hughes 2010; Read et al. 2011), but the fitness costs may also be compensated for by additional mutations (Schrag et al. 1997; Levin et al. 2000; Maisnier-Patin et al. 2002; Gagneux et al. 2006; Andersson and Hughes 2010; Perron et al. 2010). While the same arguments apply to phage resistance, fitness of bacteria surviving the phage treatment might have decreased over time if bacteria–phage coevolution resulted in bacteria continually evolving costly resistance (Buckling et al. 2006; Forde et al. 2008; Poullain et al. 2008).

**Figure 2:**
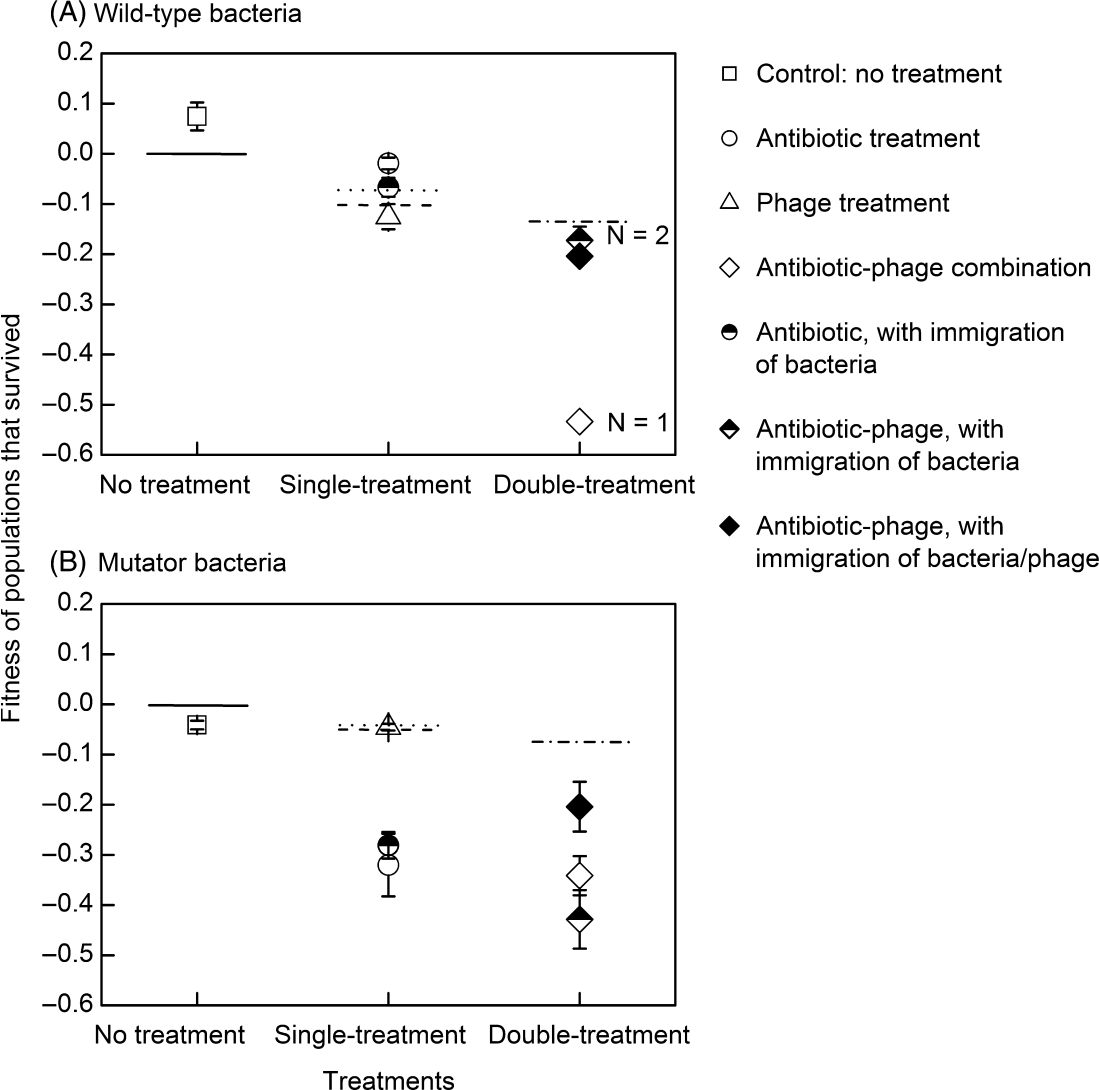
Selection coefficients (difference in fitness from the ancestor) of populations that survived different treatments, measured in antibiotic-free and phage-free environment. Data show mean (±SE) of six (unless indicated) randomly chosen populations. The solid line segments (with a *y*-axis value of 0) indicates a null hypothesis *S* value; dash (−0.106 in A and −0.047 in B), mean fitness value of the first-generation antibiotic-resistant mutants; dot (−0.069 in A and −0.044 in B), the first-generation phage-resistant mutants; and dash-dot (−0.136 in A and −0.078 in B), the first-generation double-resistance mutants.

For our bacteria, both kanamycin resistance and phage resistance conferred significant fitness costs: *S* values (difference in fitness from the ancestor) of the first-generation resistant mutants were negative (*P* < 0.05). At the end of the experiment, the wild-type bacteria from the control environment had fitness higher than the ancestor, with *S* values > 0 (*P* = 0.045; Fig. 2A). Fitness of populations surviving the antibiotic environment was higher than that of the first-generation antibiotic-resistant mutants (*P* = 0.001) and indifferent from that of the ancestor (*P* = 0.161). Fitness of phage-treated populations was marginally nonsignificantly lower than that of the first-generation phage-resistant mutants (*P* = 0.075; Fig. 2A) and significantly lower than the ancestor (*P* = 0.004). Only one population survived the combined antibiotic–phage treatment, with a very low fitness value (Fig. 2A; note that no statistical test was carried out to compare this population with the other types because of the small sample size).

Fitness of the mutator populations from each type of environment was lower than that of the ancestor, with *S* being negative (*P* < 0.01; Fig. 2B). Phage-treated populations had fitness indifferent from those from the control environment (*P* = 0.706), while populations in environments with the antibiotic (antibiotic or combined antibiotic–phage treatment) had much lower fitness (*P* < 0.01; Fig. 2B). The phage-treated populations had fitness indifferent from the first-generation phage-resistant mutants (*P* = 0.827), while populations from the antibiotic environment had fitness lower than the first-generation antibiotic-resistant mutants (*P* = 0.007), and populations from antibiotic–phage microcosms had fitness lower than the first-generation double-resistance mutants (*P* = 0.001).

## Discussion

Here, we addressed whether a combination of a phage and an antibiotic could prove effective in limiting resistance evolution in experimental *P. fluorescens* populations and hence drive the populations extinct. Neither phage nor antibiotic alone was a very effective antimicrobial agent. The phage (SBW25Φ2) could not control bacterial growth: all wild-type bacterial populations under the phage treatment survived (Fig. 1A) presumably because of rapid evolution of bacterial resistance to the phage (Buckling and Rainey 2002), although there were fitness costs associated with the resistance when measured in the absence of the phages (Fig. 2A). The antibiotic (kanamycin) treatment had a better control on bacterial survival: only 50% of wild-type populations survived (Fig. 1A), but the surviving populations showed little fitness cost (Fig. 2A). A strong synergism was found between the phage and the antibiotic, with only one of 24 populations under the combined treatment evolving resistance (Fig. 1A), presumably because of the requirement for multiple resistance mutations in the same genome to survive both phage and antibiotic attack; the surviving population also had very low fitness (Fig. 2A).

Immigration of resistance alleles from source populations, in addition to *de novo* mutation, is likely to play a crucial role in resistance evolution. In our experiment with the wild-type strain, bacterial immigration from the control (antibiotic free and phage free) environment increased the chance of resistance evolution under the antibiotic treatment, consistent with previous work (Perron et al. 2007), but not under the combined antibiotic–phage treatment (Fig. 1A). This is not surprising as immigrant populations from the control environment were likely to contain some mutants with antibiotic resistance (the spontaneous mutation rate to kanamycin resistance was approximately 10^−7^ per cell per generation, and the carrying capacity of each microcosm was >10^8^ cells), but there was little chance of mutations conferring resistance to both phages and kanamycin being present in the same individual. By contrast, immigration of bacteria/phage (from the phage-treatment environment) greatly increased the chance of resistance evolution under the combined antibiotic–phage treatment (Fig. 1A); the bacteria in the source environment had experienced selection from the phages and thus should have a fairly high probability to contain individuals with double resistance. Note that we found no evidence that phages evolving in the source microcosms in the absence of antibiotic selection showed greater infectivity than phages evolving in the sink microcosms in the presence of the antibiotic (Data S1; Figure S2). The results imply that a combined antibiotic–phage therapy might be quite robust against immigration of bacteria from a reservoir where antibiotics and the specific therapeutic phages were absent. However, rational use is as important for phage therapy as for antibiotic therapy: environmental contamination with phages used for therapeutic purposes may diminish the efficacy of combination therapy of antibiotics and phages.

Phages, unlike antibiotics, may evolve novel counter-defense strategies to overcome bacterial resistance, at a rate that researchers developing antibiotics can never hope to replicate (Alisky et al. 1998; Thiel 2004; Brockhurst et al. 2007; Pirnay et al. 2011). Phages coevolving with host bacteria may or may not be effective at reducing bacterial population sizes, depending on the relative rates of evolution of bacteria and phages (Forde et al. 2008; Poullain et al. 2008). Nevertheless, such coevolving phages are likely to drive the bacteria to continuously evolve novel defense strategies and hence to suffer increasing fitness costs (Buckling et al. 2006; Forde et al. 2008). This is also confirmed by our work with the wild-type bacteria: fitness of populations surviving the phage treatment or the combined antibiotic–phage treatment decreased over time (Fig. 2A). Therefore, where bacteria acquire resistance to, and thus survive, phage (or combined antibiotic–phage) treatment, the frequency of resistant mutants may decline when antimicrobial agents are absent.

High mutation rates in bacteria have been shown to result in very rapid evolution of resistance (Chopra et al. 2003; Denamur et al. 2005; Henrichfreise et al. 2007; Perron et al. 2010), and this was also the case in our experiment: even the combined antibiotic–phage treatment could not prevent resistance evolution in the mutator SBW25*mutS* (Fig. 1B). This constructed mutator has a very high mutation rate to phage resistance (Figure S1); it may have evolved very quickly to outpace the phage and thus rendered the phages unable to persist (Data S1), consistent with previous work of this system (Pal et al. 2007). However, fitness of the mutator bacteria significantly decreased during the selection experiment, particularly in the presence of the antibiotic (Fig. 2B). It is probable that deleterious mutations accumulated very quickly in the mutator, with the effect of these deleterious mutations enhanced in the presence of antibiotic resistance mutations (i.e., synergistic epistasis). Similarly, an increase in the effect of deleterious mutations has also been observed in the presence of phage resistance mutations (Buckling et al. 2006). Our results support the view that mutators may not able to persist on long-term timescales in nature (de Visser 2002).

Humans often adopt either a chemical or a biological treatment protocol, but not both in combination, to combat ‘harmful’ organisms such as insect pests or weeds (including those introduced to new habitats and becoming invasive species). In many cases, the biocontrol agents (e.g., parasitoids attacking herbivorous insects) can be negatively affected by the chemical agents (e.g., chemical pesticides); hence, there is antagonism rather than synergism between the biocontrol and the chemical control approaches. For bacterial control, however, nature provides us a group of special organisms, bacteriophages, which may work together with antibiotics to limit bacterial resistance evolution. While resistance to antibiotic cocktails may occur less frequently, phage–antibiotic combinations clearly have the following advantages: (i) cross-resistance to phages and antibiotics should be lower than that to multiple antibiotics and (ii) there is a potentially endless supply of therapeutic phages, whereas functional classes of antibiotics are finite.

## Data archiving statement

Data for this study are available in Supporting Information to this article on the journal’s website.

## Supporting Information

**Data S1.** Supporting methods and further description of results.

**Figure S1.** Mutation rates of the wild-type and mutator strains to kanamycin resistance and phage resistance.

**Figure S2.** Infectivity of phages from wild-typebacteria microcosms under the antibiotic-phage treatment withimmigration of bacteria/phage (treatment *vii*), and theirrespective source populations.

**Figure S3.** Growth of the wild-type bacteria under different treatments over time, measured as optical density.

**Figure S4.** Growth of the mutator bacteria under different treatments over time, measured as optical density.
