# Supplementary material for: Phages limit the evolution of bacterial antibiotic resistance in experimental microcosms
Source: Evol Appl. 2012 Jan 13;5(6):575–82. doi: 10.1111/j.1752-4571.2011.00236.x (PMC3461140; doi:10.1111/j.1752-4571.2011.00236.x)
Supplement: Supplementary file 2 [file eva0005-0575-SD2.doc]

**Supporting Information**

**Data S1. Supporting methods and further description of results**

**Mutation rates**

We used fluctuation tests to estimate bacterial mutation rates. For either the wild-type or the mutator strain, six microcosms were inoculated with ~10^2^ bacterial cells and grown overnight. Final bacterial density was determined by plating dilutions on KB agar plates and counting the number of colony forming units (CFUs) after 48 h culture at 28 °C. The number of antibiotic-resistant mutants in the final cultures was estimated by plating 50 µL of each culture on 0.1KB agar plates complemented with kanamycin (8.0 mg L^-1^); and for the number of mutants resistant to the ancestral phage, adding ~10^6^ (for the wild-type strain) or ~10^4^ (for the mutator) bacterial cells into 100 µL of phage suspensions (with ~10^8^ ancestral phage particles) and 10 min later plating onto KB agar plates. We also examined the starting bacterial populations to ensure that there was no pre-existing resistant mutant. The MSS maximum-likelihood method was used to calculate mutation rate . From these assays we randomly chose six antibiotic-resistant colonies and six phage-resistant colonies, and measured mutation rate to antibiotic resistance of the phage-resistant isolates, and mutation rate to phage resistance of the antibiotic-resistant isolates.

Mutation rate to kanamycin resistance was ~10^-7^ per cell per generation for both the ancestral SBW25 and the phage-resistant SBW25 isolates (resistant to the ancestral phage); and mutation rate to phage resistance was ~10^-7^ for both the ancestral SBW25 and the kanamycin-resistant SBW25 isolates (Fig. S1). Compared with the wild-type bacteria, the mutator had a ~10-fold higher mutation rate to kanamycin resistance (~10^-6^ for both the ancestral SBW25*mutS* and the phage-resistant SBW25*mutS* isolates), and a ~10^4^-fold higher mutation rate to phage resistance (~10^-3^ for both the ancestral SBW25*mutS* and the kanamycin-resistant SBW25*mutS* isolates; Fig. S1). Thus there is no evidence for cross-resistance against kanamycin and the phage.

**Effect of phage immigration on bacteria under the combined antibiotic-phage treatment**

When bacterial populations under the combined antibiotic-phage treatment received immigration of bacteria/phage from antibiotic-free environment, the immigrant phages may be more infective than the resident phages (as the antibiotic environment might constitute a stressful habitat for the bacteria, and bacteria-phage coevolution is typically slower in stressful habitats); and thus show a negative impact on the resident bacteria. To test for such an effect, phages from microcosms under the combined antibiotic-phage treatment with immigration of bacteria/phage (treatment *vii*) were compared with those from the source microcosms. Infectivity of a phage population on a bacterial population was determined by streaking 20 independent bacterial colonies (with a colony of the ancestral bacteria as a reference) across a line of phage (20 μL) that had previously been streaked and dried on a KB agar plate. A colony was scored as susceptible if there was inhibition of growth by the phage. Phage infectivity was estimated as the proportion of susceptible colonies . The infectivity of phage from a sink microcosm and its source microcosm can be compared by measuring the infectivity of the two phage populations on bacteria from both the sink and the source microcosms. We intended to compare phage infectivity for microcosms of both the wild-type and mutator bacteria at transfer 5 and 9. However, we found that the phage failed to grow in the mutator microcosms within 5 transfers, and in the wild-type microcosms with OD < 0.05 the bacteria were undetectable and the phage failed to grow (see below); therefore we were unable to compare phage infectivity for these microcosms. We therefore worked on the wild-type microcosms under treatment *vii* with OD ≥ 0.05: four microcosms at transfer 5 and six randomly chosen microcosms at transfer 9. Infectivity of phage was compared between these microcosms and their source microcosms.

We found that, at both transfer 5 and 9, phages from the sink microcosms (wild-type bacteria under treatment *vii*) did not differ from those from the source microcosms in infectivity (Fig. S2; ANOVA with arcsine-transformed phage infectivity as the response variable and phage source and bacteria source as categorical explanatory variables and selection line as a random factor, *P* > 0.1 for either the effect of phage or bacteria source, or the interaction effect). This implies that the antibiotic environment did not constitute a habitat that slows the rate of coevolution between (antibiotic-resistant) bacteria and phages.

**Bacterial and phage density**

In addition to measuring OD of all microcosms at all time points, we directly measured bacterial and/or phage density in a subset of microcosms. Bacterial density was measured by plating 50 μL of diluted cultures onto KB agar plates and counting the number of CFUs after 48 h culture at 28°C. To extract phage from cultures, we added 20 μL of chloroform to 100 μL of cultures, vortexed to lyse the bacteria and centrifuged at 15 800 *g* for 2 min to pellet the bacterial debris, leaving a suspension of phages in the supernatant. Phage density was estimated by spotting 10 μL of diluted phage suspension onto a lawn of the ancestral bacteria on KB agar plates and counting the number of plaque forming units (PFUs) after 24 h culture at 28 °C.

For the wild-type bacteria, six microcosms under the antibiotic-phage treatment with immigration of bacteria/phage (treatment *vii*) with OD < 0.05 were chosen at transfer 5 for the purpose of measurement of phage infectivity (see above). We found that in these microcosms both bacterial density (< 40 CFUs mL^-1^) and phage density (< 10^3^ PFUs mL^-1^) were very low. At transfer 7 we randomly chose up to six microcosms with OD < 0.05 for each treatment, and measured bacterial density (and phage density if appropriate), and found that, in all cases but one, bacterial density was < 40 mL^-1^, and phage density, < 10^3^ mL^-1^. We also measured phage densities in randomly chosen microcosms with phage introduced that had an OD ≥ 0.05, finding that in all cases phage density was > 10^5^ mL^-1^. This suggests that in microcosms with OD < 0.05 bacteria were almost completely eliminated, and phages failed to grow (each microcosm with phage treatment received ~10^2^ ancestral phage virions at each transfer, and the input phage virions would have contributed to a phage density of ~10^3^ mL^-1^).

For the mutator bacteria microcosms (all of which had OD ≥ 0.05), six microcosms under the antibiotic-phage treatment with immigration of bacteria/phage (treatment *vii*) were randomly chosen at transfer 5 for the purpose of measurement of phage infectivity. However, we found that phage density in all the six microcosms and their source microcosms was very low, < 10^3^ mL^-1^, suggesting that phages in these microcosms had failed to grow. At transfer 7, six microcosms were randomly chosen for each treatment with phage introduced (treatment *iii*, *iv*, *vi* and *vii*), of which the phage density was measured. In all cases the phage density was < 10^3^ mL^-1^. This is likely to be explained by the mutator bacteria evolving resistance much rapidly than phages evolving infectivity. Note that phages could grow to higher densities on the ancestral mutator bacteria (> 10^7^ mL^-1^).

**Literature Cited**

Buckling, A., and P. B. Rainey. 2002. Antagonistic coevolution between a bacterium and a bacteriophage. Proceedings of the Royal Society B: Biological Sciences **269**:931-936.

Forde, S. E., J. N. Thompson, and B. J. M. Bohannan. 2004. Adaptation varies through space and time in a coevolving host–parasitoid interaction. Nature **431**:841-844.

Lopez-Pascua, L. D. C., M. A. Brockhurst, and A. Buckling. 2010. Antagonistic coevolution across productivity gradients: an experimental test of the effects of dispersal. Journal of Evolutionary Biology **23**:207-211.

Luria, S. E., and M. Delbruck. 1943. Mutations of bacteria from virus sensitivity to virus resistance. Genetics **28**:491-511.

Sarkar, S., W. T. Ma, and G. v. H. Sandri. 1992. On fluctuation analysis: a new, simple and efficient method for computing the expected number of mutants. Genetica **85**:173-179.

Vogwill, T., A. Fenton, A. Buckling, M. E. Hochberg, and M. A. Brockhurst. 2009. Source populations act as coevolutionary pacemakers in experimental selection mosaics containing hotspots and coldspots. American Naturalist **173**:E171-E176.

**Figure S1.** Mutation rates of the wild-type and mutator strains to kanamycin resistance and phage resistance (resistance to the ancestral phage).


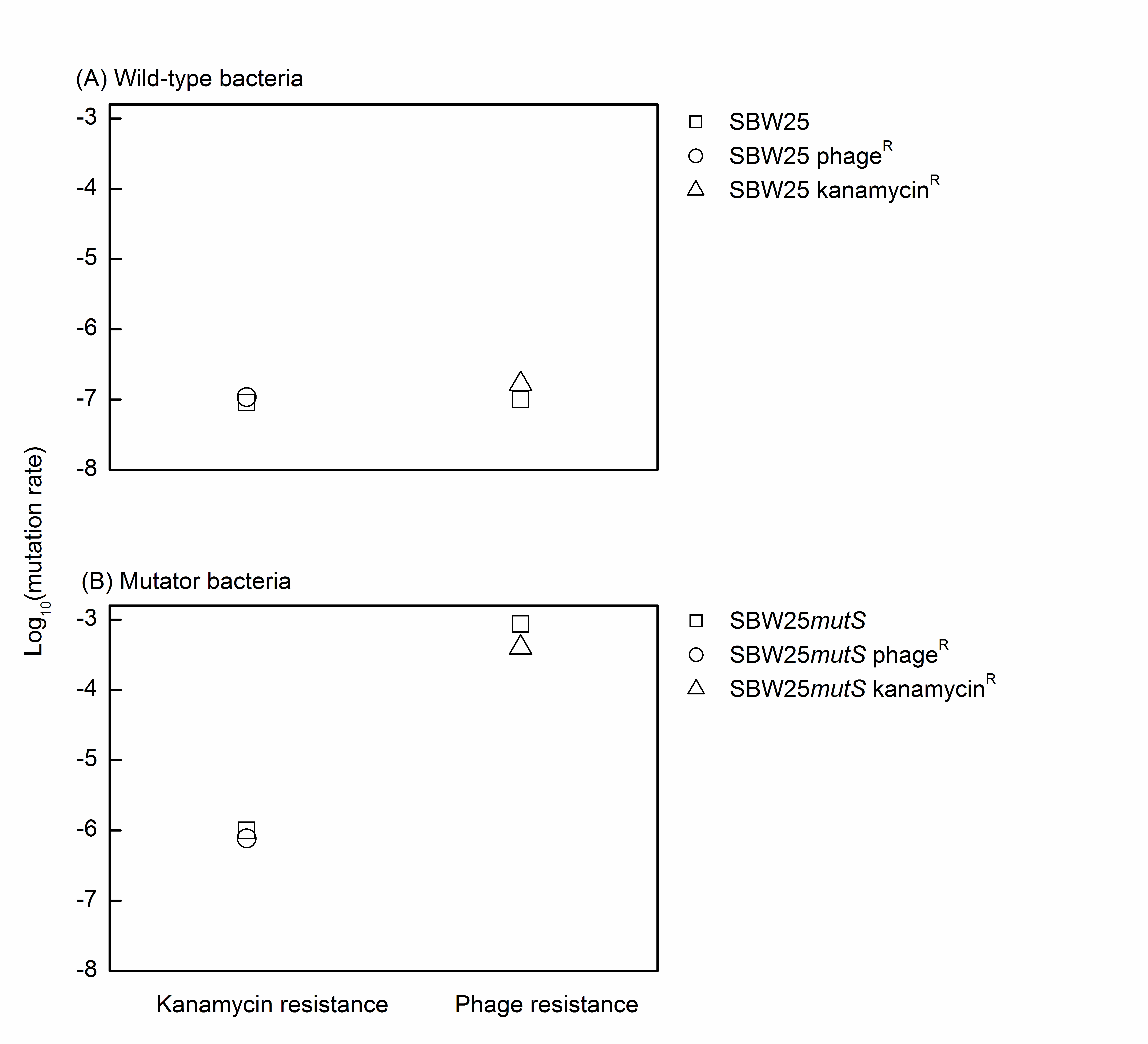


**Figure S2.** Infectivity of phages from wild-type bacteria microcosms under the combined antibiotic-phage treatment with immigration of bacteria/phage (treatment *vii*) that had an OD ≥ 0.05, (sink microcosms), and their respective source populations, measured against bacteria from both sink and source microcosms. Data show mean ±SE (N = 4 in A and N = 6 in B).


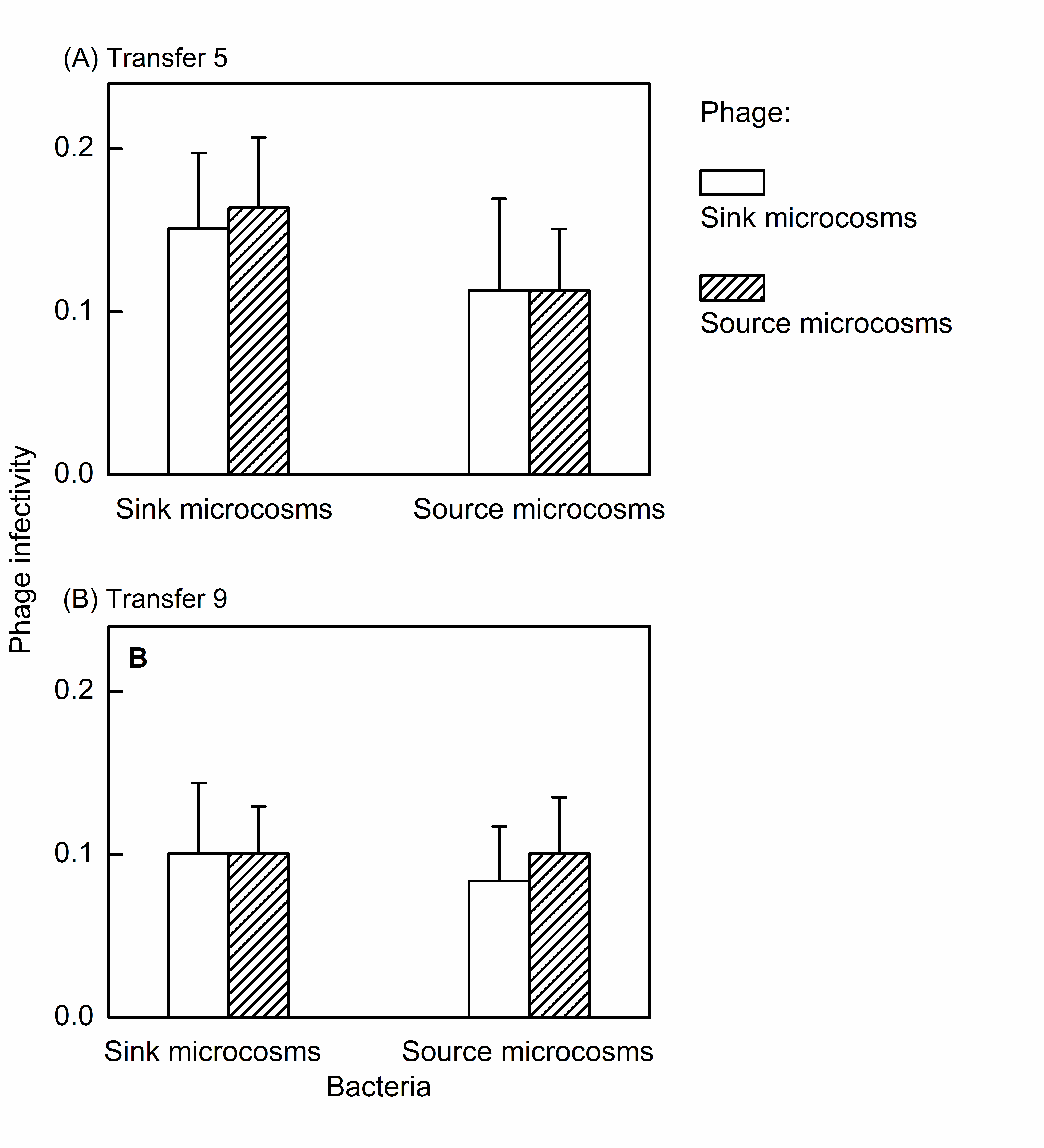


**Figure S3.** Growth of the wild-type bacteria under different treatments over time, measured as optical density (OD 600 nm). Each line represents an independent population.





**Figure S4.** Growth of the mutator bacteria under different treatments over time, measured as optical density (OD 600 nm). Each line represents an independent population.
